# A synonymous *RET* substitution enhances the oncogenic effect of an in-*cis* missense mutation by increasing constitutive splicing efficiency

**DOI:** 10.1371/journal.pgen.1007678

**Published:** 2018-10-15

**Authors:** Valeria Pecce, Marialuisa Sponziello, Giuseppe Damante, Francesca Rosignolo, Cosimo Durante, Livia Lamartina, Giorgio Grani, Diego Russo, Cira Rosaria di Gioia, Sebastiano Filetti, Antonella Verrienti

**Affiliations:** 1 Department of Internal Medicine and Medical Specialties, “Sapienza” University of Rome, Rome, Italy; 2 Department of Medical Area, University of Udine, Udine, Italy; 3 Department of Health Sciences, University of Catanzaro "Magna Graecia", Catanzaro, Italy; 4 Department of Radiological, Oncological and Pathological Sciences, “Sapienza” University of Rome, Rome, Italy; Cleveland Clinic Genomic Medicine Institute, UNITED STATES

## Abstract

Synonymous mutations continue to be filtered out from most large-scale cancer genome studies, but several lines of evidence suggest they can play driver roles in neoplastic disease. We investigated a case of an aggressive, apparently sporadic medullary thyroid carcinoma (MTC) harboring a somatic *RET* p.Cys634Arg mutation (a known MTC driver). A germ-line *RET* substitution (p.Cys630=) had also been found but was considered clinically irrelevant because of its synonymous nature. Next generation sequencing (NGS) of the tumor tissues revealed that the *RET* mutations were in *cis*. There was no evidence of gene amplification. Expression analysis found an increase of *RET* transcript in p.Cys630=;p.Cys634Arg patient compared with that found in 7 MTCs harboring p.Cys634 mutations. Minigene expression assays demonstrated that the presence of the synonymous *RET* mutation was sufficient to explain the increased *RET* mRNA level. *In silico* analyses and RNA immunoprecipitation experiments showed that the p.Cys630 = variant created new exonic splicing enhancer motifs that enhanced SRp55 recruitment to the mutant allele, leading to more efficient maturation of its pre-mRNA and an increased abundance of mature mRNA encoding a constitutively active RET receptor. These findings document a novel mechanism by which synonymous mutations can contribute to cancer progression.

## Author summary

Synonymous mutations—once considered “silent” because they do not alter the gene product’s amino-acid sequence—are now emerging as potential drivers of cancer. Our recent investigation of an aggressive medullary thyroid carcinoma (MTC) revealed a novel mechanism that could underlie such effects. The MTC analyzed harbored a somatic p.Cys634Arg mutation of the *RET* protooncogene (a well-known MTC driver). A second *RET* substitution (p.Cys630=) discovered in the germline had been considered clinically irrelevant because of its synonymous nature. Our next-generation sequencing analysis of the patient’s cancer tissues revealed that the *RET* mutations were *in cis*. We also found that *RET* mRNA levels in patient’s MTC were significantly higher than those found in seven other MTCs with various amino-acid substitutions at position 634 in *RET*. Subsequent experiments demonstrated that the presence of the synonymous mutation created new exonic splicing enhancer motifs in the mutant allele, which led to more efficient maturation of its transcript and increased expression of a constitutively active RET receptor.

## Introduction

It is now clear that synonymous mutations—single-nucleotide substitutions that do not alter the amino acid encoded by the affected codon—can play functionally relevant roles in human disease [1]. Over three decades of research have shown that these mutations can affect protein synthesis and/or function by interfering with a host of cellular mechanisms ranging from pre-mRNA splicing to protein folding [2]. Despite these advances, synonymous mutations are still largely ignored in most studies of cancer, and they continue to be filtered out from large-scale analyses of cancer genomes [3]. However, a growing body of evidence indicates that these mutations might actually play driver roles in human cancer [3,4]. Two recent studies provide evidence for the causal involvement of synonymous mutations in melanoma [5], as well as other cancer types [6].

This paper describes a series of molecular studies we conducted to explore the role played by a synonymous substitution affecting the *RET* proto-oncogene [MIM: 164761] in an aggressive case of medullary thyroid carcinoma (MTC [MIM: 155240]; http://www.omim.org). MTC is a relatively rare neuroendocrine calcitonin-secreting tumor derived from the parafollicular C-cells of the thyroid. Hereditary forms, including multiple endocrine neoplasia types 2A and 2B (MEN2A [MIM: 171400]; MEN2B [MIM: 162300]), in which MTC is associated with other endocrinopathies, are almost invariably associated with germ-line point mutations in *RET*. The membrane tyrosine kinase receptor encoded by this gene is activated by the binding of ligand—co-receptor complexes, which induces dimerization of RET proteins and autophosphorylation of intracellular tyrosine residues [7]. Gain-of-function point mutations frequently occur in RET’s cysteine-rich extracellular domain (e.g., those involving codon 634) or intracellular kinase domain (e.g., mutations at codon 918 or 804). Both result in inappropriate activation of the tyrosine kinase receptor—the former by inducing ligand-independent, disulfide-bonded homodimerization of the RET proteins, the latter by causing these proteins’ constitutive autophosphorylation [8].

Correlation has been observed between specific mutations and features of the hereditary MTC phenotype, including age at onset, tumor aggressiveness, and the presence of other endocrine tumors [9]. Somatic gain-of-function *RET* mutations appear to drive around half of all sporadic MTCs [10–12], but the genotype—phenotype correlations in these cases are less clear-cut [13].

The case we analyzed involved an apparently sporadic MTC harboring a somatic *RET* c.1900T>C mutation [p.Cys634Arg] in a previously healthy 29-year-old man (patient ID0110M). The postoperative course was characterized by rapid disease spread. Despite multiple surgical interventions and systemic vandetanib therapy [14], the patient died approximately 3 years after diagnosis (Supplemental Note). Several clinical features of this case (e.g., age at onset, aggressive disease behavior [15]) were difficult to explain solely on the basis of the somatic *RET* p.Cys634Arg driver mutation identified by Sanger sequencing. Shortly before the patient’s death, we therefore began re-analyzing DNA and RNA samples from his primary and metastatic tumors using next-generation sequencing (NGS) to identify any somatic mutations in other cancer-relevant genes. No other known somatic driver mutations were found, but the results redirected our attention to the germ-line *RET* c.1890C>T [p.Cys630=] substitution, which had been identified by the preoperative Sanger sequencing analysis and considered clinically inconsequential because of its synonymous nature. Our NGS data showed that the synonymous substitution was in *cis* with the somatic *RET* p.Cys634Arg driver mutation, and subsequent experiments demonstrated that it exerted appreciable effects on *RET* pre-mRNA splicing.

## Results

### Somatic mutational status of patient ID0110M’s tumor tissues

Using the Ion Torrent PGM NGS platform, we simultaneously sequenced 409 cancer-related genes in DNA from the patient’s preoperative blood specimen and formalin-fixed paraffin embedded (FFPE) samples of the primary and metastatic lesions. Coverage statistics and the variants identified in each tissue are reported in S1 and S2 Tables, respectively.

**Fig 1:**
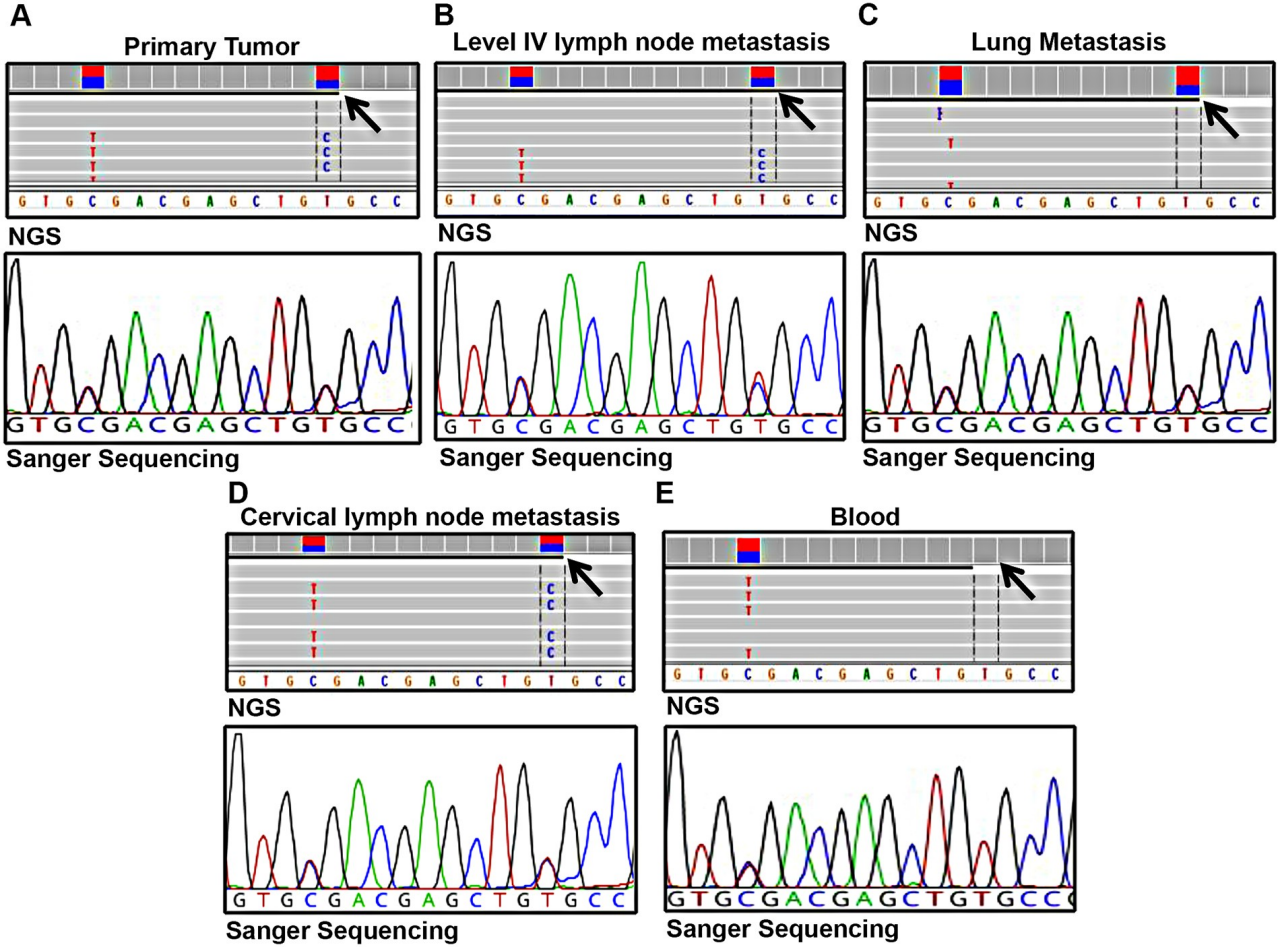
*RET* mutations harbored by patient ID 0110M. Integrative Genomics Viewer visualization and Sanger sequencing confirmation of NGS data showing the synonymous p.Cys630 = substitution and the somatic p.Cys634Arg mutation (arrows). The variations were identified in (A) the primary MTC, (B and C) two metastatic lesions removed in April 2010, and (D) a third metastatic lesion removed after discontinuation of vandetanib. The germ-line substitution was also identified in DNA from the pre-thyroidectomy blood sample (E).

As shown in Table 1, only a somatic mutation with an allele frequency (AF) ≥ 10% was shared by all four neoplastic tissue samples analyzed. This was a missense substitution (c.1900T>C [p.Cys634Arg]) in *RET* exon 11. AFs approaching 50% in all tissues (primary tumor: 37.1%; level-IV lymph node metastasis: 43.4%; lung metastasis; 32.6%; cervical lymph node metastasis: 43.4%) were consistent with mutation clonality and the absence of gene amplifications. The latter conclusion was also supported by Sanger sequencing data, which confirmed the presence of the p.Cys634Arg mutation in all four tissues and documented complete overlap between the mutant and wild-type (WT) peaks (Fig 1A–1D). NGS and Sanger sequencing findings also corroborated the presence in these tissues of the synonymous *RET* c.1890C>T (p.Cys630=) substitution documented in the germ line preoperatively (Fig 1E). The discovery that this substitution was in *cis* with the somatic p.Cys634Arg mutation led us to re-consider its possible role in the disease.

**Table 1:** Somatic missense mutations identified in the samples analyzed.

Tissue Sample (Date collected)	Blood (Jan. 2010)	Primary Tumor (Feb. 2010)	Level IV Lymph Node Metastasis (Apr. 2010)	Lung Metastasis (Apr. 2010)	Cervical Lymph Node Metastasis (Oct. 2012)
Total Variations	1114	1479	3127	8111	1252
Population Frequency Filter (MAF<0.005)					
-1000GP_EU	150	529	2171	7123	294
- ExAC_NFE	69	443	2089	7031	208
- ESP6500_Eur	69	443	2088	7031	206
ClinVar	66	439	2079	7019	206
Quality Filters					
-DP>100	48	214	1422	5699	137
- GQ>30	44	75	311	482	84
- SAF/SAR≠ 0	39	67	236	474	72
- HRUN<6	37	61	232	458	60
- AF>10%	37	54	143	255	55
Functional Filters					
- Exonic*	10	15	32	30	14
- Somatic Variants	-	5	22	22	4
- Somatic variants found in all tissues		1	1	1	1

AF: Allele frequency; DP: Depth of Coverage (total read depth at the locus); GQ: Genotype Quality (Phred-scaled marginal (or unconditional) probability of the called genotype); HRUN: Run length (the number of consecutive repeats of alternate allele in the reference genome); SAF/SAR: (Alternative allele observation on the forward strand / Alternative allele observation on the reverse strand).

* Exonic variants included nonsynonymous, stop-gain, stop-loss, splice-site variants and frameshift indels.

### Characterization of the synonymous germ-line RET c.1890C>T [p.Cys630=] substitution

Interrogation of the GnomAD database (http://gnomad.broadinstitute.org/) revealed the p.Cys630 = to be a very low-frequency single nucleotide variant (SNV), AF in the European population, 0.003% [16]. However, the c.1890C>T substitution involves codon 630 in *RET* exon 11, a common site of non-synonymous *RET* mutations known to be pathogenic (e.g., C630R, C630Y) [9]. Supek et al. maintain that synonymous mutations are significantly enriched in oncogenes, particularly those harboring activating nonsynonymous mutations, and they recurrently alter exonic splicing enhancer or silencer (ESE and ESS, respectively) motifs, causing intron retention, exon skipping, or aberrant forms of alternative splicing [6]. These considerations led us to explore the potential effects of the germ-line p.Cys630 = substitution on the quality of the *RET* transcript.

### Effects of p.Cys630=;Cys634Arg on *RET* mRNA

**Fig 2:**
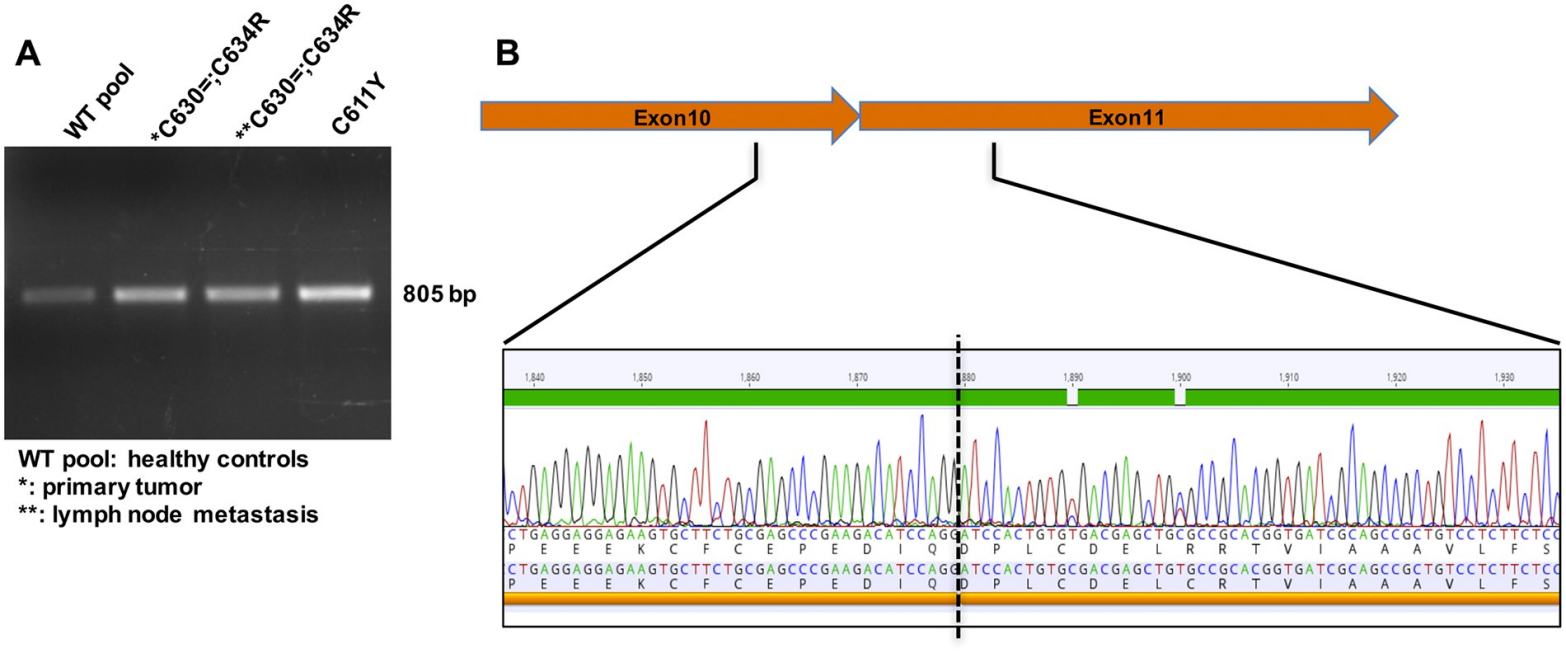
*RET* transcript sequencing and expression levels. (A) RT-PCR analysis of RET exons 10, 11 and 12 in cDNA from an FFPE sample of patient ID0110M’s primary MTC and fresh-frozen samples of the cervical lymph node metastasis (p.C630=;C634R). Findings were compared with those for a primary p.Cys611Phe MTC (p.C611Y) and for pooled cDNA from commercially available normal thyroid tissues. (B) Electropherogram of the RET exon 10–11 junction (dashed line) in cDNA from patient ID0110M’s primary tumor (shown in panel A).

We used RT-PCR to analyze *RET* mRNA quality of the region that includes exons 10, 11, and 12 in an FFPE sample of patient ID0110M’s primary MTC and a fresh-frozen sample of the cervical lymph node metastasis. The resulting amplicons were identical in length to those observed in healthy control (WT pool) and in a fresh-frozen sample of a MTC harboring a different *RET* mutation (c.1832G>A [p.Cys611Phe]) (Fig 2A). These findings argue against the presence of macroscopic alterations involving exon 11 in the *RET* transcript (e.g., retention in the mature transcript of an intronic region between exons 10 and 11, exon 11 skipping), although we cannot exclude the possibility of alternatively spliced transcripts that had already undergone nonsense-mediated decay (NMD) [17]. Sanger sequencing revealed no sequence alterations within the exon 10–11 junction in cDNA from our patient’s tumors (Fig 2B).

**Fig 3:**
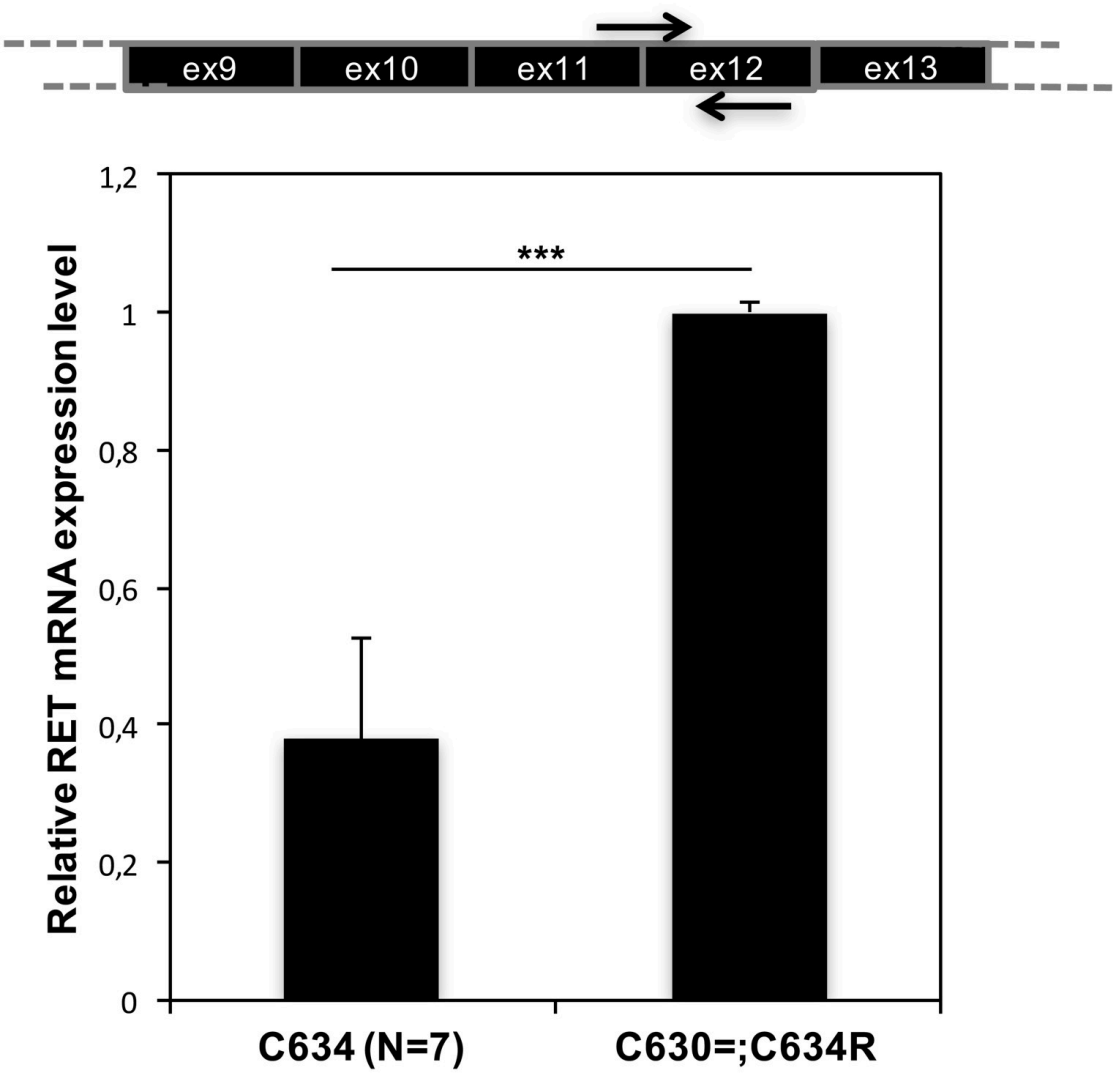
Expression of *RET* mRNA in the cervical lymph node metastasis from the p.Cys630=;Cys634Arg MTC. Results are compared with those found in 7 MTCs harboring p.Cys634Arg itself (n = 2) or similar *RET* mutations (c.1091G>C p.C634S [n = 1], c.1091G>T p.C634F [n = 1], c.1091G>A p.C634Y ([n = 2]), c.1902C>G p.C634W [n = 1]). All expression levels were analyzed by real-time PCR, RNA was isolated from fresh-frozen tissues. Results were calculated using the 2−ΔΔCt method and *GADPH* as reference control. Data reported are the means (SD) of two technical replicates. ***P = 0.00005 (t-test).

As shown in Fig 3, *RET* mRNA levels in patient ID0110M’s MTC were significantly higher than those found in seven other MTCs with various amino-acid substitutions at position 634 in *RET*, including two with the missense p.Cys634Arg mutation present in patient ID0110M’s tumor. Sanger sequencing analysis of the patient’s cervical lymph node metastasis revealed no mutations within the 1000-bp region preceding the *RET* start codon or in the 3′ UTR of *RET* transcript (S3 Table). Biologically relevant alterations involving distant enhancer regions cannot be excluded (although published data linking such mutations to MTC are currently lacking).

On the whole, these findings suggest that the p.Cys630 = substitution may exert quantitative effects on *RET* mRNA.

### Effects of increased *RET* mRNA levels on RET protein expression and activation

**Fig 4:**
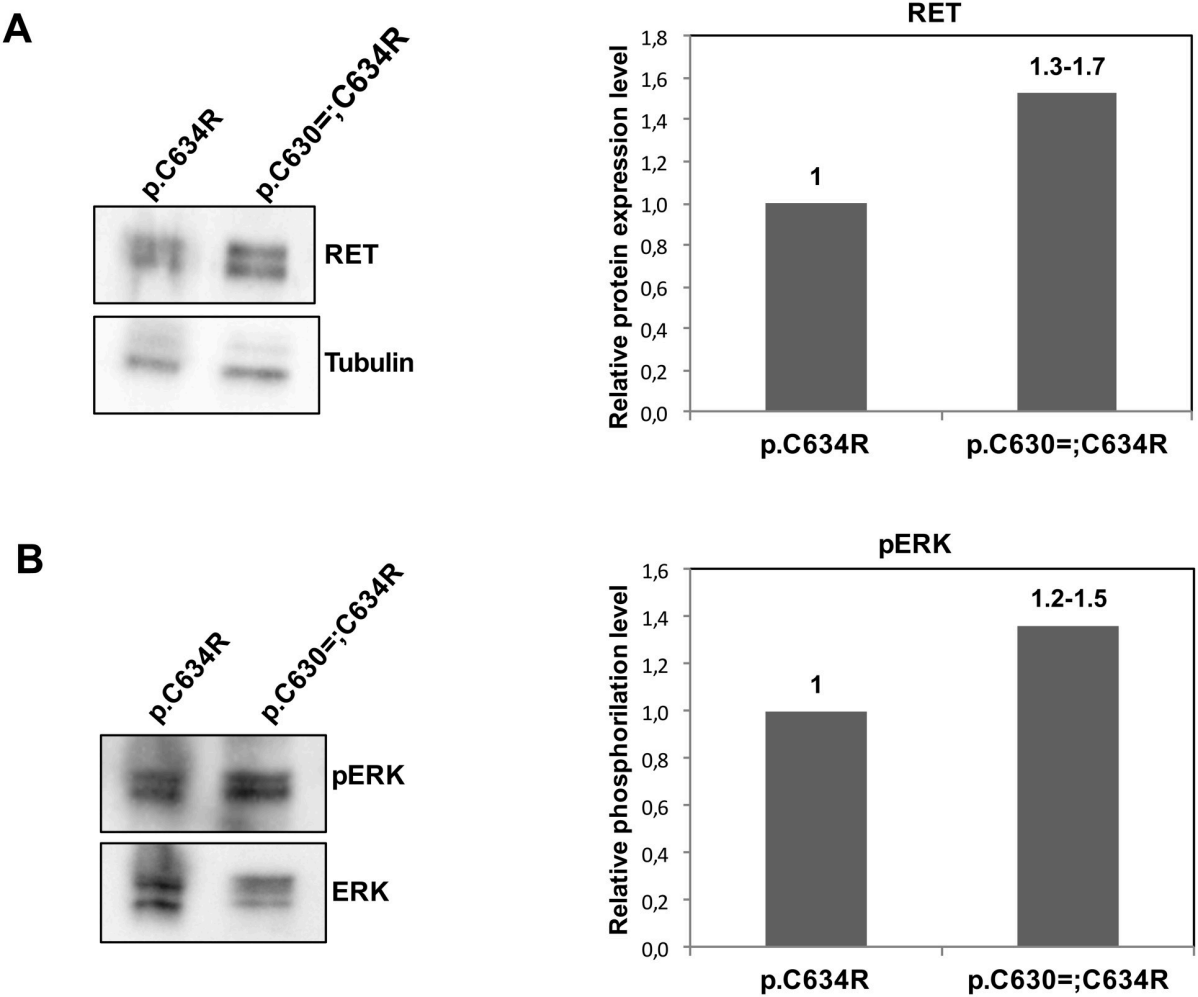
Expression and activation of RET protein in cervical lymph node metastases from p.Cys630=;Cys634Arg (patient ID0110M) and p.Cys634Arg MTCs (control). Tissues from the two cases were subjected to: (A) Western blot analysis and quantification of RET levels relative to tubulin expression. (B) Western blot analysis and quantification of phosphorylated ERK protein (pERK) relative to ERK expression. The densitometric analyses in right panels shown the mean of two technical replicates of the Western Blot in left panels, the numbers above the column represent the range obtained in the two replicates.

To further explore if the p.Cys630=;Cys634Arg quantitative effect on *RET* mRNA was translated in a more abundant RET protein, we used Western blotting to assess RET protein levels associated with the p.Cys630=;Cys634Arg phenotype (Fig 4).

Compared with a nodal metastasis from a p.Cys634Arg MTC (control), the lymph node lesion with the p.Cys630=;Cys634Arg phenotype displayed increased expression of RET protein (Fig 4A), and the latter effect was associated with increased phosphorylation of RET’s downstream target ERK (Fig 4B). Patient ID0110M’s tumor cells (with the p.Cys630=;Cys634Arg phenotype) thus appeared to contain higher levels of *RET* mRNA, and this alteration resulted in more abundant activated RET protein than that found in a p.Cys634Arg MTC.

### Effects of the synonymous substitution on the abundance of mature *RET* transcript

**Fig 5:**
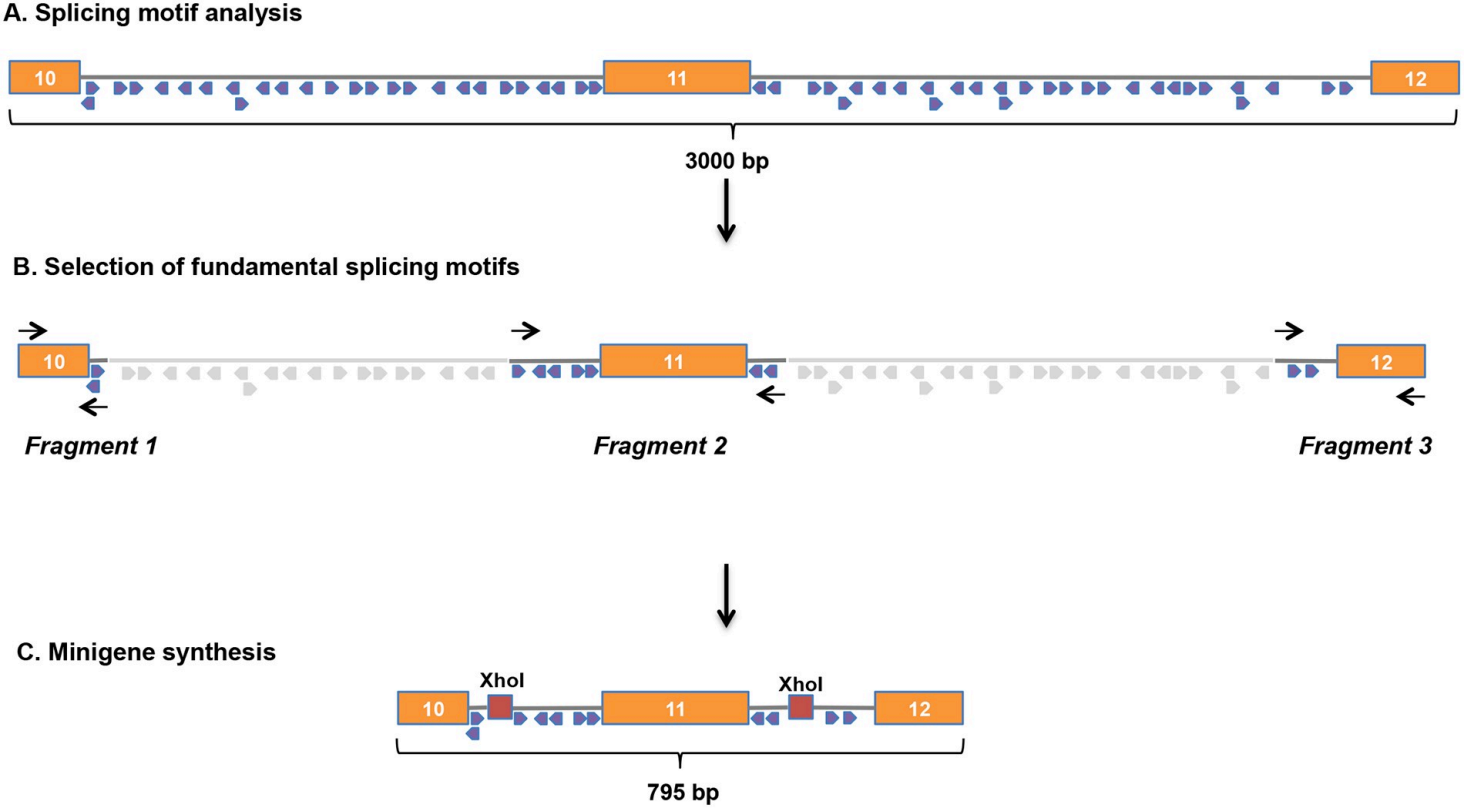
Minigene cloning strategy. (A) The 3000-bp region of RET containing exons 10, 11, and 12 (orange bars) was analyzed with Geneious 10.0.5 software to reduce the intronic sequences including only fundamental consensus splicing motifs (donor, acceptor and branch sites) (26) (B) Three regions were then chosen for amplification, each including an exon plus upstream and/or downstream flanking intronic sequences containing motifs considered fundamental for splicing based on their type and location (26). Using specific primers (black arrows), we amplified each of the three fragments from patient-derived DNA containing WT-RET sequences. Fragment 2 was also amplified from DNA containing p.Cys630=, p.Cys634Arg, or both. Amplified fragments were ligated using overhangs left by XhoI restriction enzyme digestion. (C) The ligated products, 795-bp regions containing *RET* exons 10, 11, and 12, were used to create a WT *RET* minigene and three other minigenes containing RET p.Cys630=, p.Cys634Arg, or p.Cys630=;Cys634Arg.

**Fig 6:**
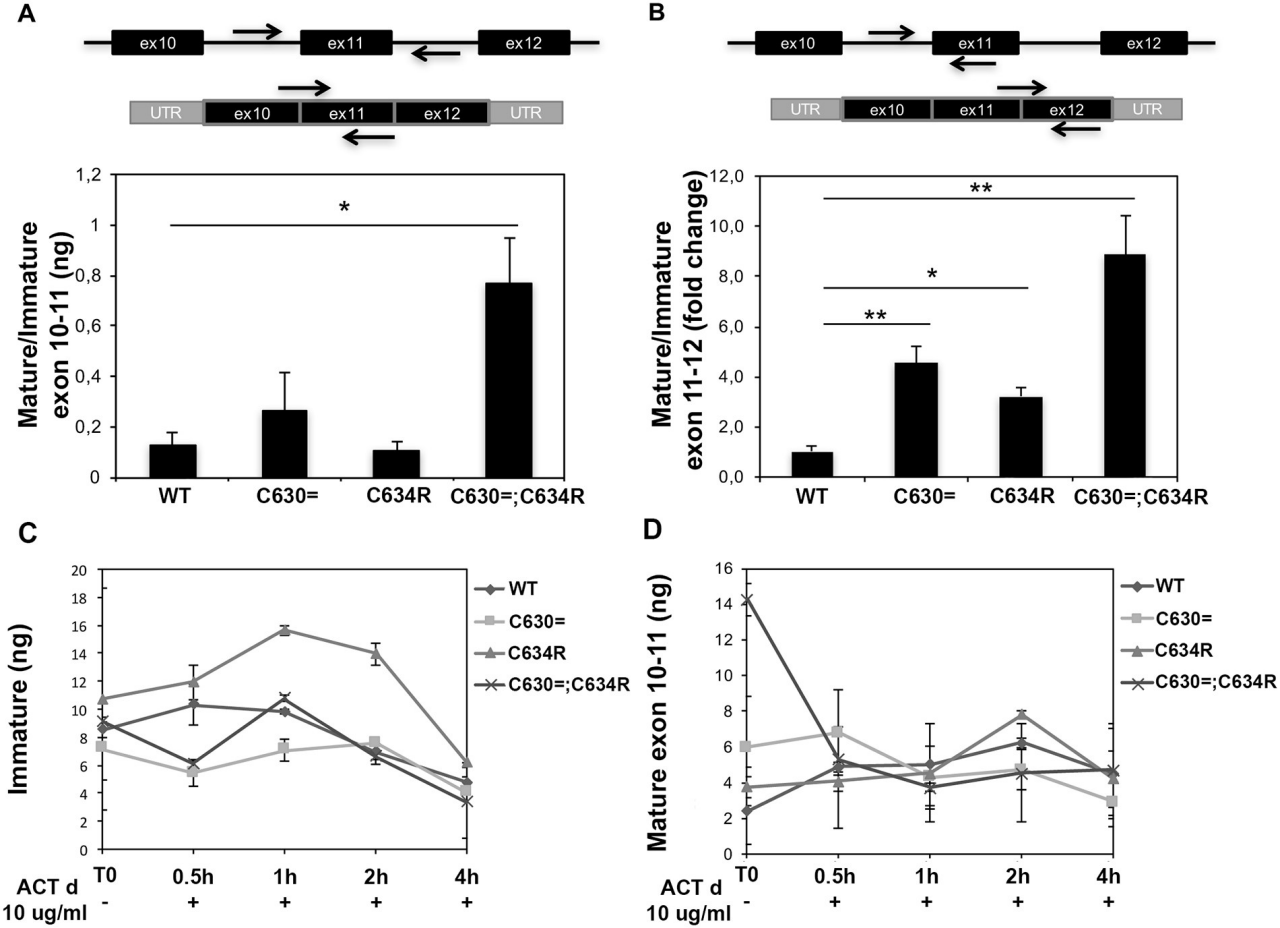
RET minigene transcript levels and stability in HeLa cells. Expression levels of mature RET transcripts 24 h after their transfection into HeLa cells (A) Real-time PCR performed on exons 10–11 junction. Schematic representation of the primers for immature and mature *RET* transcripts was reported above the panel. Expression data, obtained using standard curves, are reported as the ratio between mature and immature transcript amounts. Data are shown as the mean of two biological replicates, error bars represent S.D. *P = 0.03 (t-test). (B) Real-time PCR performed on exon 11–12 junction. Schematic representation of the primers for immature and mature *RET* transcripts was reported above the panel. Relative expression levels of mature transcripts have been expressed as 2^−ΔΔCt^ using immature transcript as normalizer and WT sample as calibrator. Data are shown as the mean of two biological replicates, error bars represent S.D. *P = 0.02 and **P = 0.019 (t-test). (C) Immature and (D) mature minigene transcript levels over time after the treatment with Actinomycin D.

To explore the causal role of the p.Cys630 = substitution in the increased abundance of mRNA *RET* transcript, we used the minigene approach, an important tool for the identification and *in vivo* analysis of regulatory elements that affect precursor RNA maturation [18]. As shown in Fig 5A–5C, we created four *RET* minigenes, each consisting of exons 10, 11, and 12 plus flanking intronic sequences with fundamental consensus splicing motifs (donor, acceptor, branch sites). Minigenes containing the WT *RET* sequence or one of the mutant sequences (p.Cys630=, p.Cys634Arg, or p.Cys630=;Cys634Arg) were transfected into HeLa cells, which do not endogenously express *RET* (S1A Fig). Twenty-four hours later, cells were harvested, and levels of immature and mature *RET* minigene transcripts were measured using two different methods, RT-PCR and real-time PCR. Expression levels of immature transcript from the four minigenes were similar to one another, but substantial differences were noted in mature transcript levels (S1B Fig). We also compared the minigenes in terms of their mature/immature *RET* transcript ratios. For this analysis, we considered the exon 10–11 and exon 11–12 junctions in mature minigene transcripts separately. As shown in Fig 6A, mature exon 10–11 junction transcript levels for the p.Cys630 = minigene were ~3-fold higher than those of the WT minigene, and the increase was even more substantial for the p.Cys630=;Cys634Arg minigene (~9-fold higher than WT transcript levels, *P* = 0.03). Similar effects were observed when we analyzed mature transcript levels for the exon 11–12 junction (Fig 6B).

Transcript levels for both minigenes harboring the p.Cys630 = substitution were always higher than those for the minigenes without this substitution. While these findings do not allow us to draw any conclusions on the possible effects on *RET* transcript levels of the p.Cys634Arg mutation, they strongly suggest that the presence of the p.Cys630 = substitution alone is sufficient to explain the increased abundance of mature *RET* transcript seen in Fig 6A and 6B. Moreover, the results of both analyses suggest a synergic effect of p.Cys630 = and p.Cys634Arg variations.

Collectively, these data suggested that the increase might be mediated by effects exerted on the maturation of minigene pre-RNA or the stability of the minigene transcripts. To explore the latter possibility, we treated the minigene-transfected HeLa cells with the transcription inhibitor, actinomycin D and monitored immature and mature transcript levels over time. No differences were observed between the four minigenes in terms of their immature or mature transcript half-lives (Fig 6C and 6D). It therefore seems more likely that the p.Cys630 = variant impacts RET expression by influencing the efficiency of maturation of *RET* pre-mRNA.

### Effects of the synonymous substitution on *RET* transcript sequence

The location of the synonymous p.Cys630 = substitution at the beginning of *RET* exon 11 (nucleotide 11) suggested that its effects on *RET* mRNA levels might be mediated by changes involving the splicing process. To explore this possibility, we used four *in silico* platforms to analyze the WT and p.Cys630=;Cys634Arg-mutated sequences of *RET* exon 11 for potential splice sites, potential branch points, and ESS and/or ESE sequences. ESEs are recognized by SR proteins, highly conserved, structurally related splicing factors that promote exon definition, directly (by increasing recruitment of the splicing machinery) or indirectly (by antagonizing the action of splicing silencers) [19]. As shown in Table 2, all four tools predicted that the p.Cys630 = substitution would create one or more new ESE motifs (recognized by SRp55 or less commonly by SF2/ASF) within exon 11 or enhance the SR protein-binding affinity of one or more ESEs already present in WT exon 11. No such changes were predicted for the region containing the p.Cys634Arg mutation (which affects nucleotide 21 of exon 11), and none of the tools predicted the creation or elimination of ESSs or any other alterations within the mutated sequence. These data suggest that the synonymous *RET* p.Cys630 = substitution increases the binding affinity of the SRp55 and SF2/ASF proteins for the mutant allele pre-mRNA, possibly enhancing recruitment of the splicing machinery to exon 11.

**Table 2:** Predicted impact of the RET p.C630 = substitution on ESE motifs.

Software	Predicted effect^a^		Location of ESE in exon 11	ESE-regulating SR Protein	SR Protein Binding Affinity Scores^b^		
	Enhanced SR prot. binding affinity of existing ESE	New ESE			WT	C630=;C634R	Software reference values
HSF	-	X	nt 6–12	SRp55	-	74.82	0–100
	X	-	nt 9–15	SF2/ASF	79.32	82.82	
ASSEDA	X	-	nt 9–15	SF2/ASF	1.5	5.0	> 0
	-	X	nt 5–11	SRp55	-	3.5	
	-	X	nt 7–13	SRp55	-	0.2	
	-	X	nt 9–15	SRp55	-	0.3	
ESE Finder	X	-	nt 7–13	SRp55	0.2	2.8	>1.9
	X	-	nt 10–16	SF2/ASF	3.0	3.6	>2.6
ESResearch	-	X	nt 7–13	-	-	New site	n.a.
	-	X	nt 9–15	-	-	New site	

*Abbreviations*: ASSEDA, automated splice site and exon definition analyses; ESE, exonic splicing enhancer; ESResearch, enhancer splicing research; HSF, human splicing finder; nt, nucleotide; n.a., not available.

^a^ Predicted effects refer to changes in the RET p.C630 = substitution compared with WT sequence.

^**b**^ Scores predictive of binding between SR protein and ESE site.

### *De novo* ESE motifs created by the p.Cys630 = substitution affect spliceosome recruitment

**Fig 7:**
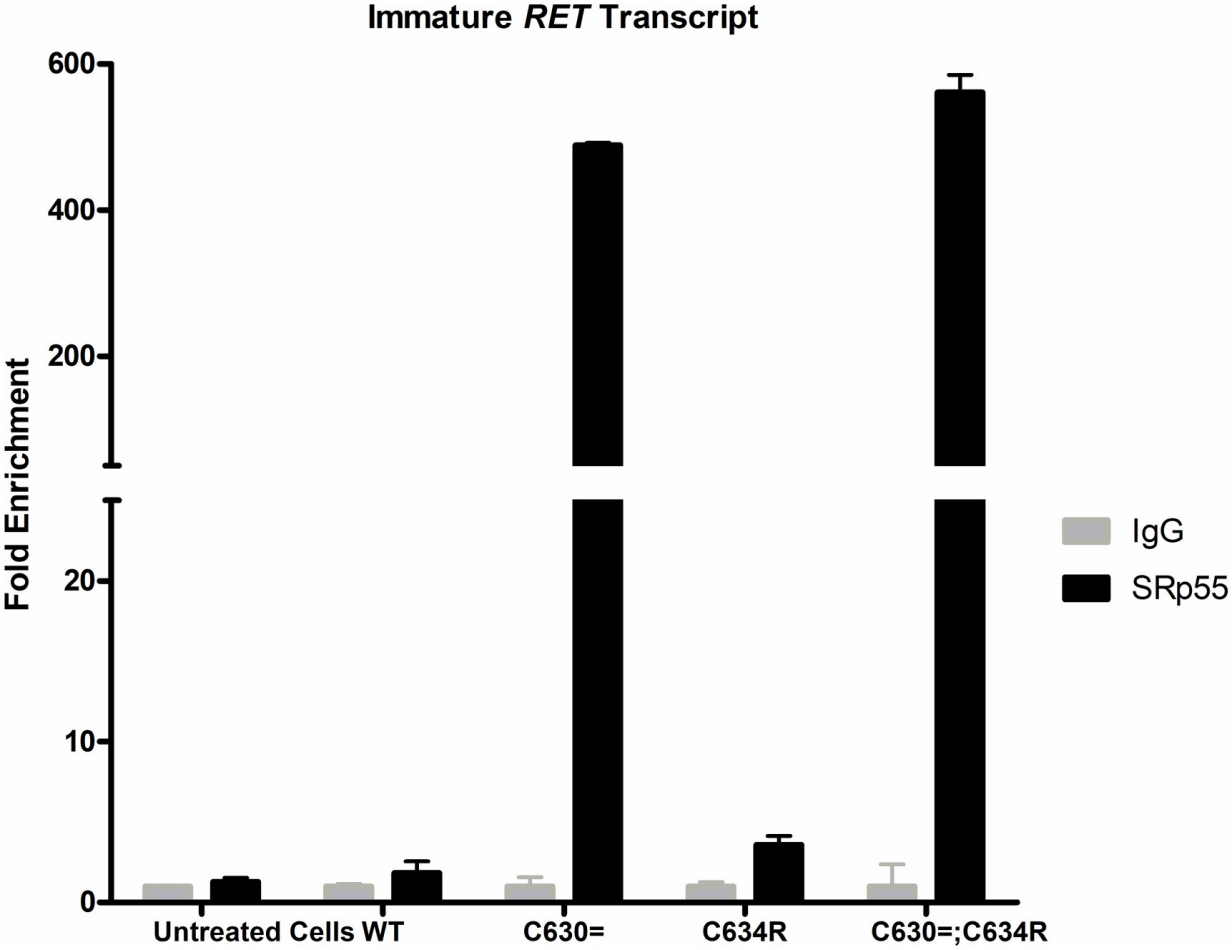
RNA IP data showing SRp55 protein interaction with immature *RET* minigene transcripts. Enrichment of extracts from HeLa cells (untreated or transfected with WT, Cys630=, Cys634Arg, Cys630=;Cys634Arg RET minigenes) compared with respective control samples (IgG). Levels of each immature transcript have been expressed as 2^−ΔΔCt^ using immature transcript levels of the respective input sample as normalizer and IgG control sample as calibrator. Results are shown for one of the two biological replicates tested. Data shown are the means of two technical replicates. Error bars represent S.D. **P = 0.0008; ***P = 0.00002 (t-test). These results were confirmed in a second independent experiment.

To assess the above hypothesis, we used RNA immunoprecipitation (RIP) to evaluate the SR protein-recruiting capacities of the immature *RET* minigene transcripts. Nuclear extracts from minigene-transfected HeLa cells were immunoprecipitated with an antibody specific for SRp55 (the SR protein most likely to recognize the new motifs, according to *in silico* predictions—see Table 2). Total SRp55-bound RNA was then isolated, reverse-transcribed, and analyzed by real-time PCR to quantify the presence of immature *RET* transcript. As shown in Fig 7, the immunoprecipitated RNA was strongly enriched for immature *RET* p.Cys630 = and *RET* p.Cys630=;Cys634Arg transcripts (488- and 561-fold, respectively, compared with the IgG-precipitated control). These findings do not directly demonstrate SRp55’s causal role in the increased levels of mature *RET* transcript. However, they are fully compatible with the hypothesis that the new SRp55-binding ESE motifs created by the synonymous p.Cys630 = substitution enhanced splicing machinery recruitment to exon 11.

## Discussion

Synonymous variants continue to be filtered out from pipelines used to analyze data from large-scale cancer genome/exome studies [20]. Consequently, they are under-reported, and their potential to drive cancer has been underestimated. Recent evidence shows, however, that these variants can indeed play functionally relevant roles in neoplastic disease [3,6,21]. Supek et al. [6] found them to be significantly over-represented in oncogenes, as compared with both cancer-unrelated genes and tumor-suppressor genes, and up to half of all oncogene-associated synonymous variants appear to be under selection. They also reported a strong association between synonymous variants in oncogenes and aberrancies involving *alternative* splicing, reflecting the ability of these substitutions to generate, alter, and/or eliminate exonic splicing regulatory motifs. To validate their *in silico* predictions, the authors used minigene expression assays to assess the effects of 12 synonymous oncogene substitutions on exon inclusion/skipping patterns in their mature transcripts. Six of the 12 were found to affect alternative splicing by creating new ESEs and/or eliminating ESSs. These changes led mainly to diminished exon skipping, which in some cases caused increased expression of full-length isoforms of the encoded protein and, consequently, enhanced oncogene activity [6].

The synonymous *RET* p.Cys630 = substitution found in our patient’s germ-line DNA involved a constitutive exon (exon 11), i.e., one fundamental for the functional integrity of the encoded protein. The complex multifactorial process regulating constitutive splicing has thus evolved to ensure that these essential exons are unambiguously recognized by the splicing machinery and included without fail in the mature transcript [22]. Not surprisingly then, mutations altering the sequences of *cis*-elements that contribute to this optimal regulatory set-up (including ESEs and ESSs) have consistently been found to cause pre-mRNA maturation that is dysfunctional [19,20,23,24].

These findings prompted our original search for qualitative alterations in *RET* mRNA in patient ID0110M’s tumor tissues. Instead, the tissues contained correctly spliced mature *RET* transcript but at higher levels than those found in control tumors. Consistently, *in silico* analyses did not predict that the presence of *RET* p.Cys630 = in exon 11 would reduce or eliminate ESE motifs in the pre-mRNA transcript from the mutant allele: instead, it was expected to create de novo ESEs and enhance the binding affinity of others for SR protein splicing regulators. These findings were also in line with our *in vitro* demonstration, compared with its WT counterpart, immature *RET* p.Cys630 = minigene transcript binds more SRp55, a protein implicated in spliceosome recruitment rather than in transcription regulation [25,26]. Therefore, instead of provoking exon-11 missplicing, the RET p.Cys630 = substitution increased levels of correctly spliced mature transcript from the mutant allele, possibly by increasing the number of molecules of pre-mRNA entering in the splicing pathway.

We used *RET* minigenes to investigate the synonymous substitution’s effect on exon 11 splicing at both its 5′ and 3’ ends. We chose to reduce intron size to more easily incorporate RET exons 10–12 into our splicing reporter construct. This design appears to have diminished the overall splicing efficiency compared with that achieved during *in vivo* transcript maturation, thereby facilitating our demonstration of the synonymous substitution’s effect on RET minigene maturation. Our *in vitro* findings led us to exclude the possibility that the p.Cys630 = substitution affects transcription or the stability of immature or mature forms of RNA. These data strengthen our suspicion that the variant’s effects were mediated by its impact on pre-RNA splicing, although they cannot exclude the involvement of other molecular mechanisms *in vivo*.

Patient ID0110M’s germ-line RET p.Cys630 = substitution occurred in *cis* with a somatic missense *RET* mutation (p.Cys634Arg), a known driver of MTC. It is difficult to speculate on the potential clinical impact of the p.Cys630 = substitution *in the absence of p*.*Cys634Arg mutation* (particularly in light of the substantial gaps in our patient’s family history [Supplementary Note]). It is reasonable to assume, however, that a genetic variant with no effects on the amino-acid sequence of the RET protein would not generate a constitutively activated RET receptor. In that case, RET signaling levels would remain dependent on ligand availability. In terms of its biological impact, the higher levels of mature transcript produced by the p.Cys630 = substitution-bearing allele may resemble those generated by gene amplification. Ciampi et al. reported *RET* amplifications in both familial and sporadic MTCs, where their presence was generally correlated with relatively poor clinical outcomes. However, with rare exceptions, these amplifications were found in tumors that also harbored a *RET* mutation. The authors therefore excluded the possibility that *RET* amplification is an alternative mechanism of *RET* gene activation, suggesting instead that it might exert potentiating effects during the transformation and/or progression of *RET*-mutated MTCs [27].

This conclusion is in line with the results of our analyses. The presence of the synonymous p.Cys630 = substitution led to more efficient maturation of transcript from the mutant *RET* allele, which also harbored the somatic p.Cys634Arg mutation. Expression of p.Cys630=;Cys634Arg allele was thereby increased (at both the transcript and protein levels) over levels that would be expected in the absence of p.Cys630=. The result was an excess of RET p.Cys634Arg monomers, which are capable of ligand-independent dimerization and autophosphorylation of the RET receptor. Such effects could conceivably augment the p.Cys634Arg mutation’s transforming capacity, but on the basis of currently available data, we cannot draw any meaningful conclusions on this possibility. Moreover, the tumor’s transforming potential could also be influenced by multiple other mechanisms, genetic (i.e., CNV or mutations in distant regulatory regions) and/or epigenetic, and we cannot exclude their involvement in our patient’s disease on the basis of the experiments conducted thus far.

Although the mechanism we hypothesize has not been previously described, it may be more common that it currently appears, given the high frequency of synonymous substitutions in oncogenes harboring activating non-synonymous mutations and their ability in this setting to increase the number of ESE motifs [3,6]. Further study is mandatory, but the analyses we have conducted thus far shed new and interesting light on the complex regulation of splicing.

## Materials and methods

All experiments performed included positive and negative controls, and commercial products were used according to the manufacturer’s instructions.

### Ethics statement

Research has been approved by our Institutional Review Board (Comitato Etico dell’azienda policlinico Umberto I). Study title: “Beyond RET: identification of the new genes involved in RET wild-type medullary thyroid cancer using Next Generation Sequencing based approach”, (Rif. 3367/25.09.14).

Written informed consent was obtained from all donors for use of their samples in genetic studies.

### Tissue collection

Neoplastic tissues were obtained with written informed consent from patients undergoing surgery for MTC at the Umberto I Hospital (Sapienza University of Rome). Two pathologists confirmed the neoplastic phenotype of all tissues based on standard histopathological criteria. Tumors were staged according to the criteria of American Joint Committee on Cancer [28].

Surgical samples of neoplastic tissue and peripheral venous blood were collected from patient ID0110M. The blood sample was used for the preoperative mutational analysis of *RET*. Sanger sequencing and NGS were used for postoperative mutational analysis of FFPE samples of the patient’s primary tumor and metastases (one to the lung, one to a level IV lymph node obtained before vandetanib treatment, one to a cervical node obtained after vandetanib treatment). FFPE samples of the primary tumor and cervical node metastasis were used to asses qualitative changes in *RET* mRNA. All FFPE were pathologist-identified samples of tumor (rather than stromal) tissue. Fresh-frozen tissue from the patient’s cervical node metastasis was used to assess qualitative changes in *RET* mRNA and protein expression. The results of both analyses were compared with those for fresh-frozen tissues from MTCs used in previous studies (21) and a non-neoplastic control pool of RNA from 64 normal thyroids (Clontech). RET protein data on patient ID0110M’s MTC were compared with those on an archived fresh-frozen sample of a lymph node metastasis from RET [p.Cys634Arg] MTC. Due to the paucity of the tissues, we performed a single protein extraction. All fresh-frozen neoplastic tissue samples had pathologist-confirmed tumor cell contents > 80%.

### Sanger sequencing

*RET* mutations were identified by Sanger sequencing of DNA from peripheral blood and FFPE samples of the primary and metastatic tumor tissues. Blood DNA was isolated using Qiagen’s QIAmp-Blood MIDI Kit; FFPE-tissue DNA using NucleoSpin Tissue Kit (Macherey-Nagel GmbH & Co.). *RET* mutational analysis was also performed on cDNA from a cervical lymph-node metastasis and from the primary tumor. Total RNA was isolated from a fresh-frozen tissue sample of the nodal metastasis using Trizol reagent (Thermo Fisher Scientific) and from FFPE tissue sample of the primary tumor using RecoverAll Total Nucleic Acid Isolation kit (Ambion). After DNase treatment, the RNA was used to generate cDNA with the High Capacity cDNA Reverse Transcription kit (Thermo Fisher Scientific). Sanger sequencing analyses of DNA and cDNA were performed as previously described [29], using the primers shown in S4 Table.

### Next-generation sequencing (NGS)

We performed NGS analysis on DNAs from peripheral blood and FFPE primary and metastatic tumor tissues. Using the Ion AmpliSeq Comprehensive Cancer Panel (Thermo Fisher Scientific), we sequenced 409 cancer driver genes. We amplified 40 ng of DNA by PCR using four premixed primer pools (Ion AmpliSeq Comprehensive Cancer Panel) and Ion AmpliSeq HiFi mix (Ion AmpliSeq Library Kit 2.0). The multiplexed amplicons were treated with a FuPa reagent to partially digest primer sequences and phosphorylate the amplicons. Adapters were ligated to digested amplicons using the Ion AmpliSeq Library Kit 2.0. To clonally amplify each DNA fragment onto the IonSphere Particles (ISPs), we performed emulsion PCR of each library on an Ion One Touch2 Instrument. Templated-ISPs were then isolated using the Ion One Touch Enrichment System. Sequencing was performed with the Ion PGM Sequencing 200 Kit version 2, using two 318 chips for each DNA sample.

### Data analysis and Variant prioritization

We analyzed data using Variant Caller v4 (Thermo Fisher Scientific). Variant caller format files were annotated with Ion Reporter 4.0 (Thermo Fisher Scientific) and wANNOVAR. Variants were called when a position was covered at least 100 times. We set the clinical sensitivity of point mutations and indels at 10%. Variant prioritization was based on population frequency, quality values, and functional consequences. Variants were filtered based on their frequency among the European-descendent population from the 1000 Genomes Project (http://www.internationalgenome.org), ESP6500SI (evs.gs.washington.edu), and ExAC datasets (http://exac.broadinstitute.org) and on clinical associations (ClinVar database) (https://www.ncbi.nlm.nih.gov/clinvar). Rare variants were defined as those with a minor allele frequency (MAF) < 0.5%. Variants classified by ClinVar as “not-pathogenic,” “probable-not-pathogenic,” “drug response,” or “other” were excluded. High-quality variants were those with a depth of coverage (DP) of ≥100, genotype quality (GQ) scores of ≥30, a minimum alternate allele frequency of 10% (AF≥10%), and absence of both strand bias (SAF/SAR ≠ 0) and homopolymer regions (HRUN<6). Finally, variants were prioritized based on their genomic location, with exclusion of intronic, intergenic, ncRNA-intronic, and UTR variants. Synonymous variants were excluded by the default functional filter. Exonic, splicing, stop-gain, stop-loss, and frameshift insertion and deletion variants were retained for further evaluation.

### RNA analysis

Total RNA was isolated from FFPE slides with NucleoSpin tissue (Macherey Nagel), from fresh-frozen tissues with Trizol reagent (Thermo Fisher Scientific), and from transfected cells with RNeasy Mini Kit (Qiagen). After DNase treatment and purification (precipitation with ethanol and sodium-acetate), RNAs were quantified with Nanodrop 2000 (Thermo Fisher Scientific) and used to synthesize cDNA with the High Capacity cDNA Reverse Transcription kit (Thermo Fisher Scientific). Qualitative changes in *RET* mRNA and *RET* expression in tissue samples and minigene-transfected cells were assessed with RT-PCR (performed with AmpliTaq Gold [Thermo Fisher Scientific] according to a standard protocol) and real-time PCR. The RETcDNAF8 and RETcDNAR12 primers used for RT-PCR assays are reported in S4 Table. Minigene expression in transfected cells was assessed by RT-PCR with Immature F2 and Immature R1 primers for amplification of immature minigene transcript and MatureF1 and RET rs11 primers for mature transcript (S1A Fig). Amplification was repeated 25 times for the immature transcripts and 40 times for mature transcripts. Primer sequences are in S4 Table.

Real-time PCR analyses were done with a 7900HT Fast Real-Time PCR System, and SDS 2.3 software (both from Thermo Fisher Scientific) was used to calculate Ct values [30]. TaqMan Universal Master Mix was used for quantitative analyses with TaqMan Gene Expression Assays-on-Demand using a standard protocol. Results were calculated using the 2^−ΔΔCt^ method and normalized to the calibrator sample. In particular, TaqMan Gene Expression Assays-on-Demand Hs01120021_m1 and Hs99999905_m1 (Thermo Fisher Scientific) were used to quantify *RET* and *GADPH* (reference control) expression, respectively, in MTC samples. *RET* expression in patient ID0110M was used as the calibrator. The TaqMan Gene Expression Assay-on-Demand Hs01120021_m1 (Thermo Fisher Scientific) was used to quantify mature minigene exon 11–12 junction transcript levels. Immature minigene transcript levels were quantified with Taqman Gene Expression Assay AIWR4LA (Thermo Fisher Scientific) designed with the Taqman Gene Expression design tool (https://www.thermofisher.com/order/custom-genomic-products/tools/gene-expression/) and the minigene sequence as a template. The assay targeted the ligation sites (i.e. XhoI restriction sites, see “RET minigene cloning strategy and plasmid isolation” paragraph). Thermo Fisher Scientific certifies the high amplification efficiency (of 90–110%) of all Taqman gene expression assays. Immature minigene transcripts encoded by minigenes were used as reference control and the WT sample (ratio mature/immature) as a calibrator.

The SensiMix SYBR kit (Bioline) was used with specific primer pairs to quantify minigene expression levels and the half-lives of immature and mature minigene transcripts using a standard protocol. A standard curve was used to assess primer efficiency. Slopes, Y-intercepts, and R^2 values were -3.836, 22.087, and 0.975 for Immature F2 and Immature R1 and -3.253, 111.48, and 0.645 for Mature F1 and RETrs11. Levels of immature and mature minigene transcripts in transfected HeLa cell extracts were expressed as nanograms of cDNA, the ratio of mature to immature transcript levels, or percentage of mature transcripts.

Custom Taqman Gene Expression Assay AIWR4LA (Thermo Fisher Scientific) was used to quantify immature minigene transcript levels in RNA immunoprecipitate and IgG-precipitated extracts (negative controls). Results were normalized to input samples and expressed relative to IgG control.

### Protein extraction and analysis

Total proteins were extracted on ice from fresh-frozen tissues and quantified using the Bradford method. The lysis buffer contained TrisHCl (pH 7.4, 50 mM), NaCl (150 mM), Triton (1% v/v), EDTA (20 mM), phenylmethylsulfonyl fluoride (2 mM), protease and phosphatase inhibitors (Pierce), leupeptin (2 μg/ml), and glycerol (10% v/v). For Western blot analysis, 30 μg of proteins separated by SDS-PAGE, transferred to polyvinylidene fluoride membranes, and probed with the following primary antibodies (all from Cell Signaling Technology and all used at 1:1000 dilution): anti-RET (C31B4), anti-Tubulin (sc-8035), anti-Erk1/2 (cat.# 137F5), and anti-phospho-ERK1/2 (Thr202-Tyr204) antibody (cat.# 4370). Secondary antibodies (anti-mouse SC-2005 and anti-rabbit SC-2004) were HRP-conjugated and used at 1:5000 dilution. Membranes were incubated with ECL reagent (Clarity Western, Bio-rad), and chemiluminescence was quantified with Bio-Rad’s Chemidoc MP Imaging system (Bio-rad). Band intensity was analyzed with the system’s Image Lab Software and the results normalized to that of controls (tubulin bands for RET; ERK bands for phospho-ERK bands). Experiments were repeated twice.

### RET minigene cloning strategy and plasmid isolation

We used PCR to amplify three *RET* gene fragments containing exon 10, exon 11, or exon 12, each with flanking intronic sequences. Fragments were amplified using AmpliTaq Gold (Thermo Fisher Scientific). Primer sequences are shown in S4 Table (RETex10_F, RETex10_XhoI_R, RETex11_XhoI_F, RETex11_XhoI_R, RETex12_XhoI_F, RETex12_SalI_R). Fragment 1, which included exon 10 plus the first 23 nucleotides (nt) of intron 10, and fragment 3, which comprised the last 99 nt of intron 11 and all of exon 12, were both amplified from patient ID0110M’s DNA containing the WT *RET* allele. Fragment 2 contained the last 128 nt of intron 10, all of exon 11, and the first 20 nt of intron 11. Four versions were created: one with the WT sequence (amplified from the DNA used for fragments 1 and 2); the second with the p.Cys630 = substitution (amplified from germ-line DNA from patient ID0110M); the third with the p.Cys634Arg mutation (amplified from DNA isolated from an MTC harboring this mutation); and the fourth containing both of the latter variations (amplified from somatic DNA isolated from patient ID0110M). Amplified fragments were cloned into a pGEM-T Easy Vector (Promega) linked by the XhoI restriction enzyme sites. The sequences of all fragments were verified by Sanger sequencing. The construct was subcloned into a pcDNA3.3-TOPO TA vector (Thermo Fisher Scientific) and used to transform JM109 competent cells (Promega), using the heat-shock procedure. The transformed cells were plated on Luria broth (LB) agar containing 100 mg/mL ampicillin and incubated overnight at 37°C. Positive colonies were suspended in 2 ml of LB supplemented with 100 mg/mL ampicillin. After 16 hours of incubation at 37°C with shaking, the bacterial mixture was used to isolate plasmids, as previously described [31].

### HeLa cell transfection and actinomycin D treatment

The day before transfection, HeLa CCL-2 cells purchased from ATCC were seeded (0.8 x 10^5^) into 6-well culture plates containing Dulbecco’s modified Eagle medium (DMEM) (Gibco by Thermo Fisher Scientific) supplemented with 10% fetal bovine serum (FBS), penicillin (100 U/mL), and streptomycin (100 μg/mL) and incubated at 37°C in a 5% CO2 incubator. Two hours before transfection, the medium was replaced with antibiotic- and FBS-free DMEM. The starved cells were then transfected with *RET* minigenes (2 μg) using OPTI-MEM medium and Lipofectamine 3000 (both by Gibco, Thermo Fisher Scientific). After a 24-h incubation, the transfected cells were harvested, RNA was isolated, and cDNA was generated and assayed to quantify minigene expression, as described above (see “RNA analysis”).

For the treatment with Actinomycin D (Thermo Fisher Scientific), we plated HeLa cells (0.4x10^5^) into 12-well culture plates and, as described above, four minigenes were transfected in Hela cells. After 24-h from transfection; we treated HeLa cells with 10 μg/ml [32] of Actinomycin D (Thermo Fisher scientific) for 30 minutes, 1-, 2- and 4-hours. Cells were harvested at established time point trough enzymatic method (0.5% trypsin) after two washes in PBS, in order to preserve only viable cells. Transfected and treated cells were pelleted at 1000 rpm for 5 minutes. Total RNA was isolated from transfected and treated cells with Qiagen’s RNeasy Mini Kit. RNA was treated with DNase and cDNA was generated using the High Capacity cDNA Reverse Transcription kit (Thermo Fisher Scientific). The half-lives of mature transcript minigenes were assessed as described in “RNA analysis” paragraph.

### Splicing motif analysis

Four platforms were used to identify altered splicing motifs and splicing factor binding sites in exon 11 of *RET*: the Human Splicing Finder (http://www.umd.be/HSF3/); ESResearch (http://ibis.tau.ac.il/ssat/ESR.htm); Automated Splice Site And Exon Definition Analyses (ASSEDA) (http://splice.uwo.ca); and ESE Finder (http://krainer01.cshl.edu/cgi-bin/tools/ESE3/esefinder.cgi?process=home).

The results were obtained analyzing the entire sequence of exon 11 WT or with p.C630=;C634R variations in all queries of the four platforms.

### RNA immunoprecipitation (RIP)

We treated *RET* minigene-transfected HeLa CCL-2 cells with 1% formaldehyde for 10 minutes at room temperature to crosslink RNA-protein complexes. The cells were washed in glycine (125 mM) to stop the reaction, harvested, and resuspended in PBS diluted 1:1 with nuclear isolation buffer (NIB) consisting of sucrose (1.28 M), Tris-HCl pH 7.5 (40 mM), MgCl2 (20 mM), and Triton X-100 (4%). To lyse the nuclear membranes, we resuspended the nuclear pellet in RIP buffer containing KCl (150 mM), Tris pH 7.4 (25 mM), EDTA (5 mM), DTT (0.5 mM), NP-40 (0.5%), RNAase inhibitor (100 U/ml), and protease inhibitors. One tenth of the total volume of nuclear lysate was reserved for use as an input control. Chromatin was needle-sheared, antibody was added to the solution (anti-SRp55 antibody [Millipore] for RIP samples, anti-IgG antibody [Millipore] for negative control samples), and samples were incubated at 4°C with gentle rotation to enhance antibody-protein binding. One hour later, magnetic protein G beads (Dynabeads, Thermo Fisher Scientific) were added and the samples re-incubated for 1h at 4°C with gentle rotation. Unbound material was removed by two washes with RIP buffer and one in PBS. The de-crosslinking reaction was performed at 70°C for 10 minutes. RNA and protein were isolated with TRIzol reagent (Thermo Fisher Scientific). cDNA was generated with High Capacity cDNA Reverse Transcription kit (Thermo Fisher Scientific).

Real-time PCR was performed as described above in the paragraph “RNA analysis”.

### Statistical analysis

Statistical significance was assessed with the Student’s t test, and results were considered significant when P values were <0.05.

## Supporting information

S1 Supplementary Note—Case report (DOCX)

S1 Fig *RET* minigene transcript. (A) Representative gel from one of three RT-PCR experiments. Black arrows indicate immature and mature transcript bands. (Lower mature transcript bands represent concatemers of primers.) (B) The amount of each transcript (mature and immature) measured by real-time PCR is reported as separated bars; numbers above bars indicate the percentage of mature transcript in each sample.
(TIF)

S1 Table NGS coverage statistics. (DOC)

S2 Table List of Prioritized Variants in cervical lymph node metastasis. (XLS)

S3 Table SNPs found in -1000bp and 3’UTR regions of RET gene in patient ID0110M. (DOCX)

S4 Table Primer sequences. (DOC)
