# Supplementary material for: A synonymous RET substitution enhances the oncogenic effect of an in-cis missense mutation by increasing constitutive splicing efficiency
Source: PLoS Genet. 2018 Oct 15;14(10):e1007678. doi: 10.1371/journal.pgen.1007678 (PMC6201961; doi:10.1371/journal.pgen.1007678)
Supplement: S1 Supplementary Note—Case report — (DOCX) [file pgen.1007678.s001.docx]

**Supplementary Note – Case report**

January-February 2010: Patient ID0110M (age 29) presented with a history of tender, nodular swelling in the left lateral neck. There was no known family history of thyroid disease or cancer. Fine-needle aspiration cytology was consistent with metastatic medullary thyroid cancer (MTC). Total thyroidectomy with central and left laterocervical lymphadenectomy was performed. The left thyroid lobe contained a single tumor (maximum diameter: 3.5 cm) histologically diagnosed as MTC, which had infiltrated the perithyroid adipose tissue and metastasized to the lymph nodes (AJCC stage: pT3, pN1b, Mx).

March-April 2010: Genetic testing revealed the presence of a somatic *RET* mutation (p.C634R) known to drive sporadic MTCs. A synonymous germ-line *RET* p.C630C substitution was also noted but regarded as clinically irrelevant. Post-operative imaging demonstrated persistent neck disease and distant metastases. Left upper lobectomy was performed for MTC lung metastases and mediastinal and level IV lymph node metastases were also resected (pM1, stage IV).

October 2010: Imaging studies revealed multiple recurrences in the left paratracheal tissue, laterocervical lymph nodes (bilateral), mediastinum, lungs and its hilar lymph nodes. Surgery was excluded. Active surveillance (biochemical markers, imaging studies) continued.

July 2011: Patient met the RECIST (Response Evaluation Criteria In Solid Tumors (1) criteria for enrollment in a clinical trial of vandetanib (300 mg / day), which maintained the tumor burden at essentially stable levels with level I-II adverse effects for several months.

July 2012: After 12 months of vandetanib, ongoing surveillance documented clear progression of the disease in the neck, lung, and mediastinum, and the drug was discontinued.

October 2012: Radiotherapy to the lung for right upper lobe lesions, followed by lymphadenectomy for involvement of the right jugular lymph nodes and left laterocervical lymph nodes. All lesions were histologically confirmed to be MTC metastases.

July 2013: Brain metastases were discovered and treated with radiotherapy. The lung disease worsened rapidly, and the patient died in October 2013.

**Reference:**

1. Eisenhauer EA, Therasse P, Bogaerts J, Schwartz LH, Sargent D, Ford R, et al. New response evaluation criteria in solid tumours: Revised RECIST guideline (version 1.1). Eur J Cancer [Internet]. 2009;45(2):228–47. Available from: http://dx.doi.org/10.1016/j.ejca.2008.10.026
