# Supplementary figures and images for: A synonymous RET substitution enhances the oncogenic effect of an in-cis missense mutation by increasing constitutive splicing efficiency
Source: PLoS Genet. 2018 Oct 15;14(10):e1007678. doi: 10.1371/journal.pgen.1007678 (PMC6201961; doi:10.1371/journal.pgen.1007678)

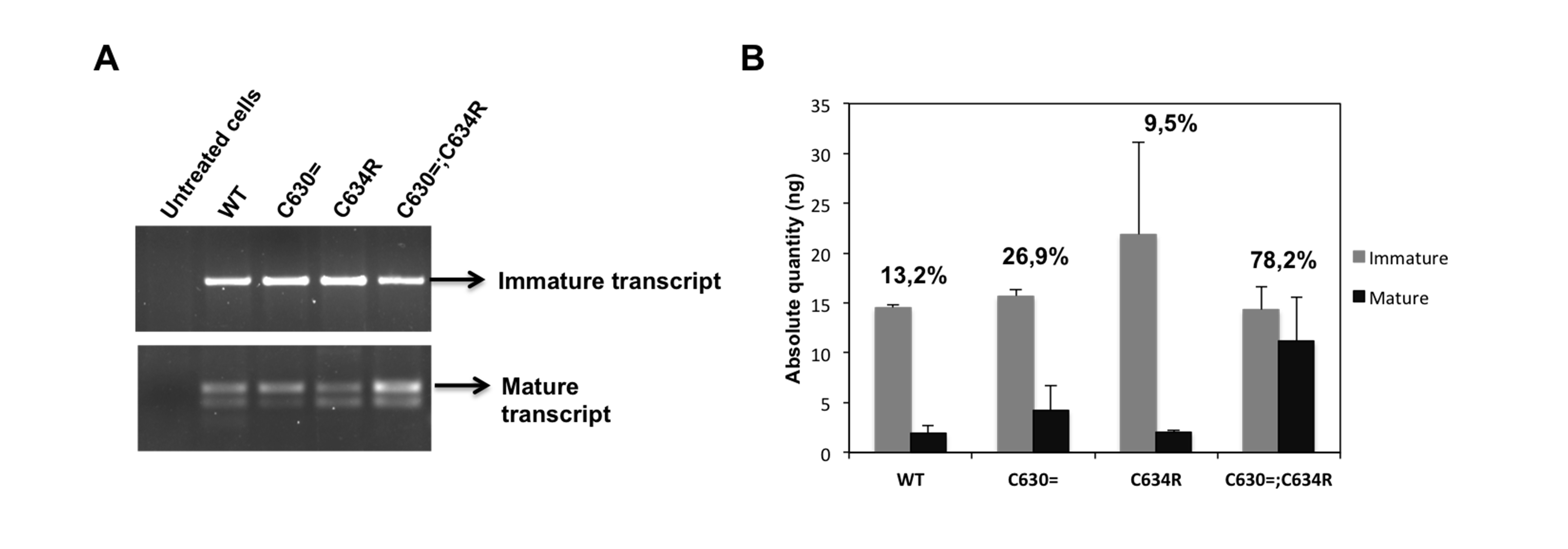

Supplement: S1 Fig — (A) Representative gel from one of three RT-PCR experiments. Black arrows indicate immature and mature transcript bands. (Lower mature transcript bands represent concatemers of primers.) (B) The amount of each transcript (mature and immature) measured by real-time PCR is reported as separated bars; numbers above bars indicate the percentage of mature transcript in each sample. (TIF) [file pgen.1007678.s002.tif]
