# Supplementary material for: A synonymous RET substitution enhances the oncogenic effect of an in-cis missense mutation by increasing constitutive splicing efficiency
Source: PLoS Genet. 2018 Oct 15;14(10):e1007678. doi: 10.1371/journal.pgen.1007678 (PMC6201961; doi:10.1371/journal.pgen.1007678)
Supplement: S1 Table — (DOC) [file pgen.1007678.s003.doc]

**Table S1. NGS coverage statistics**

| **Tissue Samples**  **(Date collected)** | | **Blood**  **(Jan. 2010)** | **Primary Tumor**  **(Feb. 2010)** | **Level IV Lymph Node Metastasis**  **(Apr. 2010)** | **Lung Metastasis**  **(Apr. 2010)** | **Cervical Lymph Node Metastasis**  **(Oct. 2012)** |
| --- | --- | --- | --- | --- | --- | --- |
| Number of mapped reads | 10,358,613 | | 7,884,291 | 11,342,547 | 9,703,577 | 11,086,681 |
| Percent reads on target (%) | 99.01 | | 97.83 | 97.16 | 96.95 | 98.86 |
| Average base coverage depth | 663.8 | | 486.4 | 669.7 | 566.5 | 704.5 |
| Uniformity of base coverage (%) | 94.02 | | 93.48 | 84.21 | 86.54 | 92.36 |
| Target base coverage at 1x (%) | 99.70 | | 99.73 | 99.52 | 99.57 | 99.60 |
| Target base coverage at 20x (%) | 98.46 | | 98.15 | 96.54 | 96.95 | 98.02 |
| Target base coverage at 100x (%) | 95.54 | | 93.27 | 87.67 | 88.09 | 94.40 |
| Target base coverage at 500x (%) | 60.30 | | 39.88 | 48.84 | 41.73 | 60.84 |
