# Supplementary material for: A synonymous RET substitution enhances the oncogenic effect of an in-cis missense mutation by increasing constitutive splicing efficiency
Source: PLoS Genet. 2018 Oct 15;14(10):e1007678. doi: 10.1371/journal.pgen.1007678 (PMC6201961; doi:10.1371/journal.pgen.1007678)
Supplement: S3 Table — (DOCX) [file pgen.1007678.s005.docx]

| **dbSNP** | **Genotype** | **MAF** | **ClinVar_ Clinical Significance** |
| --- | --- | --- | --- |
| rs2435366 | C/C | T=0.2418/1211 (1000 Genomes) | NA |
|  |  | T=0.1525/4440 (TOPMED) |  |
| rs10900296 | G/G | A=0.2632/1318 (1000 Genomes) | With Benign allele |
|  |  | A=0.1914/5574 (TOPMED) |  |
| rs2075912 | C/C | T=0.1969/20451 (ExAC) | With Benign allele |
|  |  | T=0.2450/1227 (1000 Genomes) |  |
|  |  | T=0.1467/1907 (GO-ESP) |  |
|  |  | T=0.1355/3946 (TOPMED) |  |
| rs2565200 | C/C | T=0.2346/1175 (1000 Genomes) | NA |
|  |  | T=0.1208/3516 (TOPMED) |  |
| rs2742240 | T/T | A=0.2476/1240 (1000 Genomes) | NA |
|  |  | A=0.1406/4095 (TOPMED) |  |
| rs2742241 | A/A | G=0.2528/1266 (1000 Genomes) | With Benign allele |
|  |  | G=0.1444/4204 (TOPMED) |  |
| rs76759170 | G/A | A=0.0353/177 (1000 Genomes) | With Likely benign allele |
|  |  | A=0.0419/1221 (TOPMED) |  |
| rs3026785 | T/C | C=0.0375/188 (1000 Genomes) | Likely benign, protective |
|  |  | C=0.0452/1315 (TOPMED) |  |

**Table S3. SNPs found in -1000bp and 3’UTR regions of RET gene in patient ID0110M**
