# Supplementary material for: A synonymous RET substitution enhances the oncogenic effect of an in-cis missense mutation by increasing constitutive splicing efficiency
Source: PLoS Genet. 2018 Oct 15;14(10):e1007678. doi: 10.1371/journal.pgen.1007678 (PMC6201961; doi:10.1371/journal.pgen.1007678)
Supplement: S4 Table — (DOC) [file pgen.1007678.s006.doc]

**Table S4. Primer sequences**

| **Primer Name** | **Sequence** |
| --- | --- |
| RETex10_F | GGGGCAGCATTGTTGGGGGA |
| RETex10_XhoI_R | CTCGAGGGTCCCGGCCGCCACC |
| RETex11_XhoI_F | CTCGAGTCCATGGCCACTTCCC |
| RETex11_XhoI_R | CTCGAGTGCCCCGCAGGGACCC |
| RETex12_XhoI_F | CTCGAGTTGTCCATGGGGCCTC |
| RETex12_SalI_R | GTCGACCTTTCAGCATCTTCAC |
| ImmatureF2 | GGGACCCTCGAGTCCATGGCC |
| ImmaureR1 | ATGGACAACTCGAGTGCCCCG |
| MatureF1 | GAAGACATCCAGGATCCACTG |
| RETcDNAF8 | CCAAGTGTGCCGAACTTCAC |
| RETcDNAR11 | GCAAACTTGTGGTAGCAGTGG |
| RETcDNAR12 | AAGGCCGTTGCCTTGACCAC |
| RETf11 | CCATGAGGCAGAGCATAGC |
| RET P1F | CTGGCTTTTCCTCCTCAAG |
| RET P1R | GGAAGAGAGGCAGAATAG |
| RET P2F | TCACAAACTGCCCTTCCAG |
| RET P2R | GTGTAGGAGCTCAGTGCG |
| RET P3F | ACCCCGCTCCTCCCCAAG |
| RET P3R | CACTCACCTTTGCCTAGCAG |
| RETrs11 | GCAGTGGATGCAGAAGGC |
